# Mesoscopic bar magnet based on ε-Fe_2_O_3_ hard ferrite

**DOI:** 10.1038/srep27212

**Published:** 2016-06-07

**Authors:** Shin-ichi Ohkoshi, Asuka Namai, Takehiro Yamaoka, Marie Yoshikiyo, Kenta Imoto, Tomomichi Nasu, Shizuka Anan, Yoshikazu Umeta, Kosuke Nakagawa, Hiroko Tokoro

**Affiliations:** 1 Department of Chemistry, School of Science, The University of Tokyo, 7-3-1 Hongo, Bunkyo-ku, Tokyo 113-0033, Japan; 2 Hitachi High-Tech Science Corporation, Kanagawa Science Park, R&D Business Park Bldg., C-1F, 3-2-1 Sakado, Takatsu-ku, Kawasaki-shi, Kanagawa 213-0012, Japan; 3 Division of Materials Science, Faculty of Pure and Applied Sciences, University of Tsukuba, 1-1-1 Tennodai, Tsukuba, Ibaraki 305-8577, Japan

## Abstract

Ferrite magnets have a long history. They are used in motors, magnetic fluids, drug delivery systems, etc. Herein we report a mesoscopic ferrite bar magnet based on rod-shaped ε-Fe_2_O_3_ with a large coercive field (>25 kOe). The ε-Fe_2_O_3_–based bar magnet is a single crystal with a single magnetic domain along the longitudinal direction. A wide frequency range spectroscopic study shows that the crystallographic *a*-axis of ε-Fe_2_O_3_, which corresponds to the longitudinal direction of the bar magnet, plays an important role in linear and non-linear magneto-optical transitions, phonon modes, and the magnon (Kittel mode). Due to its multiferroic property, a magnetic-responsive non-linear optical sheet is manufactured as an application using an ε-Fe_2_O_3_–based bar magnet, resin, and polyethylene terephthalate. Furthermore, from the viewpoint of the large coercive field property, we demonstrate that a mesoscopic ε-Fe_2_O_3_ bar magnet can be used as a magnetic force microscopy probe.

The first ferrite magnet, which was composed of magnetite, was discovered in Greece in the 7^th^ century BC. Today ferrite bar magnets are common and economical materials; they are used in toys, stationaries, and crafts as well as technical applications. Ferrite magnets such as Fe_2_O_3_, Fe_3_O_4_, and BaFe_12_O_19_ have contributed to various industrial products^1^,^2^,^3^,^4^,^5^,^6^,^7^,^8^,^9^. Fe_2_O_3_ has four known phases: α-, β-, γ-, and ε-Fe_2_O_3_. γ- and α-Fe_2_O_3_ are naturally existing minerals, whereas β-Fe_2_O_3_ and ε-Fe_2_O_3_ are uncommon and synthesized in the laboratory. γ-Fe_2_O_3_ has a spinel structure and is a ferrimagnet^10^,^11^,^12^. α-Fe_2_O_3_ has a corundum structure and shows weak ferromagnetism^13^,^14^,^15^,^16^,^17^,^18^,^19^,^20^,^21^. β-Fe_2_O_3_ has a bixbyite structure and exhibits antiferromagnetism^22^,^23^,^24^. ε-Fe_2_O_3_ has an orthorhombic crystal structure^23^,^25^,^26^. A pure phase of ε-Fe_2_O_3_ synthesized in 2004 was found to exhibit a large coercive field^27^. Since then, various studies on its magnetic properties have been reported^28^,^29^,^30^,^31^,^32^,^33^,^34^. The present study aims to investigate how large an ε-Fe_2_O_3_ single crystal can be grown, its magnetic property, and its magnetic domain structure. We prepare a bar magnet based on a mesoscopic ε-Fe_2_O_3_ rod and investigate its magnetic domain structure using atomic force microscopy (AFM) and magnetic force microscopy (MFM). Additionally, we measure various optical spectra over a wide frequency range from 75 GHz (wavelength *λ* = 4 mm) to 750 THz (*λ* = 400 nm) using Faraday spectroscopy, far-infrared (Far-IR) spectroscopy, terahertz (THz) time-domain spectroscopy, and non-linear magneto-optical Faraday spectroscopy. Combining the spectroscopic data with theoretical calculations for the magneto-optical transition moments, phonon modes, and the magnon, we clarify the linear and non-linear magneto-optical transitions, lattice vibrations, and the Kittel-mode magnon. Moreover, we prepare an ε-Fe_2_O_3_/resin on polyethylene terephthalate (PET) and propose a magnetically controlled non-linear optical sheet. Furthermore, since this bar magnet is resistant to external magnetic fields, we apply a mesoscopic ε-Fe_2_O_3_ rod as an MFM probe of a cantilever.

## Results and Discussion

### Morphology and crystal structure

**Figure 1:**
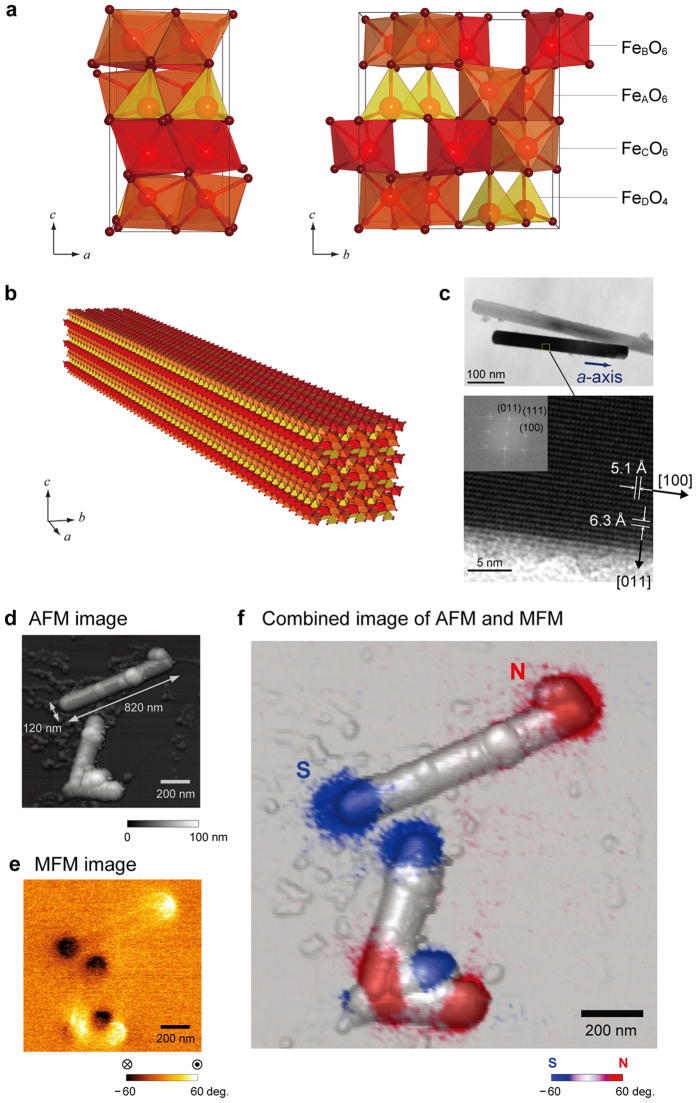
Crystal structure and AFM and MFM images of the mesoscopic ε-Fe_2_O_3_ rod. (**a**) Crystal structure of the ε-Fe_2_O_3_ unit cell (orthorhombic, *Pna*2_1_) viewed along the *b*-axis (left) and *a*-axis (right). Red, dark red, orange octahedra, and the yellow tetrahedra indicate the four nonequivalent Fe sites for Fe_A_O_6_, Fe_B_O_6_, Fe_C_O_6_, and Fe_D_O_4_, respectively. (**b**) Schematic illustration of a bar magnet based on a mesoscopic ε-Fe_2_O_3_ rod. (**c**) HRTEM images of the mesoscopic ε-Fe_2_O_3_ rod showing that the longitudinal direction is along the crystallographic *a*-axis of ε-Fe_2_O_3_. Lower figure is an enlarged image, and the inset shows the Fourier transformed image. (**d**) AFM image of the ε-Fe_2_O_3_ rods. Color scale indicates the height. One of the mesoscopic ε-Fe_2_O_3_ rods observed in the upper part of the image has longitudinal- and short-axes of 820 nm and 120 nm, respectively. (**e**) MFM image of the mesoscopic ε-Fe_2_O_3_ rods. Color scale indicates the vectors of the magnetic flux going out of (white) and into (black) the page, which correspond to the N-pole and S-pole, respectively. (**f**) Combined AFM and MFM image for the ε-Fe_2_O_3_ rods. Red and blue indicate the N-pole and the S-pole, respectively.

ε-Fe_2_O_3_ rods were prepared by combining the reverse-micelle and sol-gel techniques. (See Methods and Supplementary Information.) The scanning electron microscopy (SEM) images show that the ε-Fe_2_O_3_ rods are on the mesoscopic scale (i.e., several hundred nanometers in the longitudinal direction). The powder X-ray diffraction (XRD) pattern with Rietveld analysis indicates that the crystal structure is orthorhombic *Pna*2_1_ with lattice constants of *a* = 5.08923(16) Å, *b* = 8.7858(3) Å, and *c* = 9.4766(2) Å (Table S1). ε-Fe_2_O_3_ has four different Fe sites; the coordination geometries of Fe_A_O_6_ and Fe_B_O_6_ are distorted octahedral, Fe_C_O_6_ is regular octahedral, and Fe_D_O_4_ is tetrahedral (Fig. 1a). ε-Fe_2_O_3_ has a noncentrosymmetric crystal structure with an electric polarization along the crystallographic *c*-axis. The high-resolution transmission electron microscopy (HRTEM) measurements show that the longitudinal direction of the ε-Fe_2_O_3_ rod is the crystallographic *a*-axis (Fig. 1b,c).

### Single magnetic domain structure

ε-Fe_2_O_3_ rods with sizes near 1 μm were spread on a carbon plate for the AFM measurements as shown in the SEM images of Figure S1. Figure 1d shows the AFM topograph of an ε-Fe_2_O_3_ rod on a carbon plate. The longitudinal- and short-axes of this rod are 820 nm and 120 nm, respectively, and the height is 120 nm.

The same area was also measured by MFM. Figure 1e shows the magnetic response image. The combined AFM and MFM image shows that the north (N) and south (S) poles are at the edges of the mesoscopic ε-Fe_2_O_3_ rods (Fig. 1f), indicating that the ε-Fe_2_O_3_ rod has a single magnetic domain structure. The direction of the magnetic pole (i.e., the magnetic easy-axis) is along the longitudinal direction, which corresponds to the crystallographic *a*-axis. Such a mesoscopic scale single crystal rod with a single magnetic domain structure has not been reported for a ferrite magnet.

### Magneto-optical and optical properties

**Figure 2:**
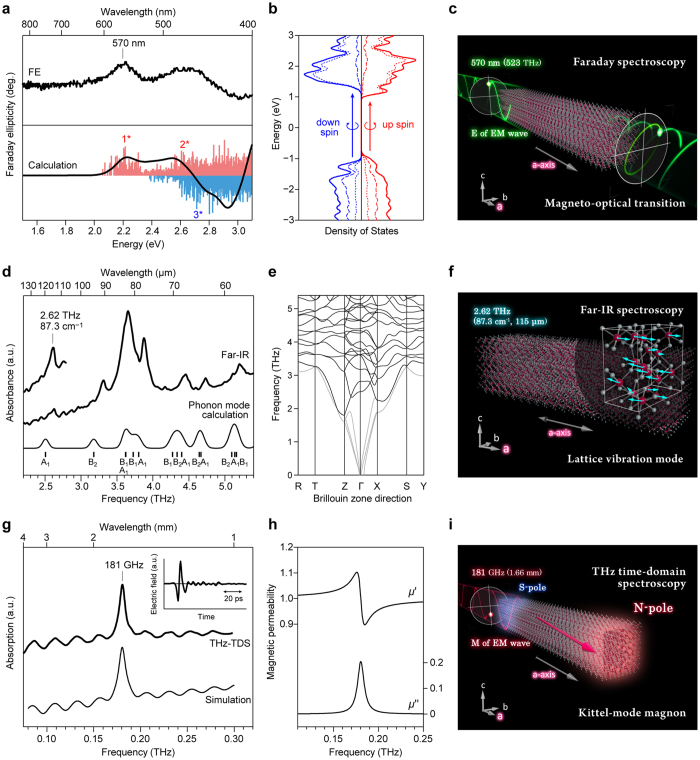
Faraday spectrum, Far-IR spectrum, and Kittel-mode magnon spectrum. (**a**) Experimentally obtained Faraday ellipticity (FE) spectrum (upper), and calculated magneto-optical transition probability by first-principles calculation (lower). Pink sticks, blue sticks, and black line in the lower panel indicate the transition probabilities of the up-spin → up-spin transitions, down-spin → down-spin transitions, and their sum, respectively. (**b**) Density of states of the electronic structure for ε-Fe_2_O_3_ obtained by first-principles calculation. Red and blue indicate the up- and down-spins, respectively, while the solid, dotted, and dashed lines indicate the density of states for the total, Fe, and O, respectively. (**c**) Linearly polarized light is converted into rotated elliptical light by the optical transition of up-spin → up-spin transition at 570 nm along the longitudinal axis (parallel to the crystallographic *a*-axis), resulting in the Faraday effect. E is the electric field of the electromagnetic (EM) wave. (**d**) Far-IR spectrum (thick lines) and the calculated spectrum (thin line) by phonon mode calculation of ε-Fe_2_O_3_. Tick marks indicate the calculated positions and the vibrational symmetries of the phonon modes. (**e**) Phonon dispersion of ε-Fe_2_O_3_. Black and gray lines indicate the optical and the acoustic phonon modes, respectively. (**f**) Atomic movement of the lowest frequency optical phonon mode of 2.62 THz (calc. 2.51 THz) with A_1_ symmetry. The Fe atoms are vibrating in the longitudinal direction of the rod (parallel to the crystallographic *a*-axis). (**g**) Kittel-mode magnon spectrum of the mesoscopic ε-Fe_2_O_3_ rod measured by THz time-domain spectroscopy (THz-TDS) (thick line) and the fitted spectrum by the Landau-Lifshitz analysis considering the interference effect between the front and back surfaces of the sample (thin line). The inset is the temporal waveform of the transmitted THz pulse. (**h**) Real (*μ*′) and imaginary part (*μ*″) of the magnetic permeability obtained by Landau-Lifshitz analysis. (**i**) Kittel-mode magnon at 181 GHz (0.181 THz) caused by the precession of the bulk magnetization (magenta arrow) around the magnetic easy-axis along the longitudinal direction (parallel to the crystallographic *a*-axis). M is the magnetic field of the EM wave.

The magneto-optical effect of a mesoscopic ε-Fe_2_O_3_ rod was measured using Faraday spectroscopy. The mesoscopic ε-Fe_2_O_3_ rod exhibits Faraday ellipticities around 570 nm and 480 nm in the visible region (Fig. 2a, upper). To understand these Faraday ellipticities, the magneto-optical transitions of ε-Fe_2_O_3_ were calculated by first-principles calculations using a Vienna ab initio simulation package. (See Methods.) The density of states of the electronic structure shows ε-Fe_2_O_3_ has a wide band gap, and the calculation well reproduces the Faraday ellipticities (Fig. 2a, lower). Both the top of the valence band and the bottom of the conduction band are polarized by up-spins (Fig. 2b).

The magneto-optical transition at 570 nm is an up-spin → up-spin transition (denoted by 1* in Fig. 2a), which is a transition from the valence band composed of the 2p orbitals of O1 and O3 to the conduction band composed of the 3d orbitals on the Fe_C_ site. The direction of the magneto-optical transition moment is mainly along the crystallographic *a*-axis (Fig. 2c and Movie 1). As for the ellipticity at 480 nm, the optical transition (denoted by 2*) is also assigned to the up-spin → up-spin transition from the 2p orbitals of O1 and O3 to the 3d orbitals of Fe_B_ and Fe_C_. On the other hand, the down-spin → down-spin transition, which is observed around 460 nm (denoted by 3*), is assigned to the optical transition from the 2p orbitals of O2, O4, O5, and O6 to the 3d orbitals of Fe_A_ and Fe_D_. The real (*ε*′) and imaginary (*ε*″) parts of the dielectric functions for each crystallographic axis were also calculated (Figure S2). The observed Faraday spectrum and the anisotropic dielectric functions show that the Faraday effect in the visible region appears when light is irradiated from the end-surface of the bar magnet in the crystallographic *a*-axis direction.

The lattice vibration mode (phonon mode) of the mesoscopic ε-Fe_2_O_3_ rod was measured using Far-IR spectroscopy. Figure 2d shows that the low-frequency range lattice vibrations are observed at 2.62 THz (87.3 cm^−1^), 3.31 THz (110.4 cm^−1^), 3.65 THz (121.8 cm^−1^), 3.87 THz (129.1 cm^−1^), 4.45 THz (148.4 cm^−1^), 4.73 THz (157.9 cm^−1^), and 5.21 THz (173.8 cm^−1^) in the frequency range of 2.2 THz (*λ* = 136 μm, 73 cm^−1^)–5.4 THz (*λ* = 56 μm, 180 cm^−1^). The Far-IR spectrum in the range of 5.4–12 THz is shown in Figure S3a.

To assign the observed Far-IR spectrum, a phonon mode calculation was carried out using the Phonon code. (See Methods.) The calculated phonon modes (Fig. 2d, lower and 2e) show similar spectral patterns to the observed Far-IR spectrum. The observed lowest frequency lattice vibration of 2.62 THz is assigned to the optical phonon mode (calc. 2.51 THz) due to the Fe atom vibration along the crystallographic *a*-axis with A_1_ symmetry (Fig. 2f and Movie 2). The calculated phonon mode spectrum between 5.4 and 12 THz and the phonon density of states are shown in Figure S3b. Table S2 lists all of the phonon modes accompanied by their symmetries and IR/Raman activities.

The Kittel-mode magnon was measured by THz time-domain spectroscopy in the millimeter wave region of 75 GHz (0.075 THz, *λ* = 4.0 mm) − 300 GHz (0.3 THz, *λ* = 1.0 mm). (See Methods.) The temporal waveform of the transmitted THz pulse is shown in Fig. 2g (inset). The Fourier-transformed spectrum shows that the mesoscopic ε-Fe_2_O_3_ rod possesses a millimeter wave absorption peak at 181 GHz (0.181 THz, *λ* = 1.66 mm) with fringe patterns of 20 GHz cycles (Fig. 2g, upper). This absorption peak is due to the resonance of the Kittel-mode magnon, originating from the precession of bulk magnetization around the magnetic easy-axis (crystallographic *a*-axis). This type of zero-magnetic-field ferromagnetic resonance is called a natural resonance. The accompanied fringe pattern is due to the interference between the front and back sample surfaces; the fringe pattern depends on the sample thickness (Fig. 2g, lower).

Considering this interference effect, the real (*μ*′) and imaginary parts (*μ*″) of the magnetic permeability (Fig. 2h) were evaluated on the basis of the Landau-Lifshitz analysis^35^,^36^ (Fig. 2i, Movie 3, and Methods). In the present ferrite bar magnet, the Kittel-mode magnon frequency is remarkably high and approaches the phonon mode frequency. The Kittel-mode magnon frequency of 0.181 THz is as high as 7% of the lowest frequency optical phonon mode of 2.62 THz. This is due to the strong magnetic anisotropy of ε-Fe_2_O_3_. Furthermore, the Kittel-mode resonance frequency in the present bar magnet at 181 GHz has almost the same value as that of the spherical ε-Fe_2_O_3_ nanoparticle at 182 GHz in our previous report^28^, indicating that the resonance frequency is independent of the material shape. Thus, the high-frequency Kittel-mode magnon originates from the intrinsic magnetic anisotropy of ε-Fe_2_O_3_, and the contribution of the shape magnetic anisotropy is insignificant.

### Polar-crystal ferrite bar magnet

**Figure 3:**
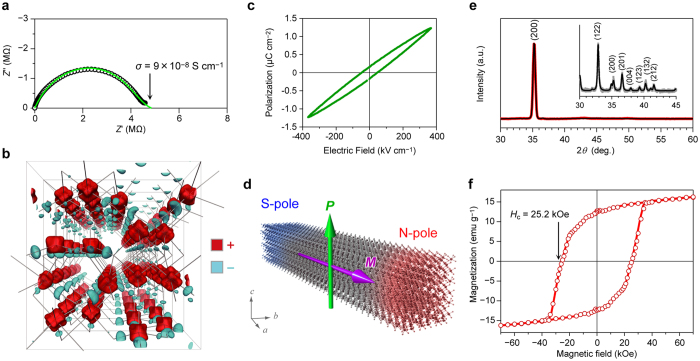
Polar-crystal ferrite bar magnet and thin film of oriented ε-Fe_2_O_3_ rods. (**a**) *Z*″ versus *Z*′ plots of the mesoscopic ε-Fe_2_O_3_ rod measured at room temperature. Green line shows the Cole-Cole arc fitting curve. (**b**) Charge polarization map of ε-Fe_2_O_3_. This figure is viewed along the *a*-axis. Green and light blue surfaces denote the positively and negatively charged densities with the isosurface levels of ±0.035 *a*_0_^−3^ (*a*_0_: Bohr radius), respectively. (**c**) Ferroelectric hysteresis loop measured at 77 K. (**d**) Schematic image of the mesoscopic ε-Fe_2_O_3_ rod with a single magnetic domain. The magnetic polarization (*M*) of ε-Fe_2_O_3_-based bar magnet is along the crystallographic *a*-axis (magenta arrow), which corresponds to the longitudinal direction of the rod, while the electric polarization (*P*) is parallel to the *c*-axis (green arrow). (**e**) XRD pattern of the film based on crystallographically oriented ε-Fe_2_O_3_ rods and XRD pattern of the nonoriented ε-Fe_2_O_3_ rods (inset). Red line, gray line, and black lines indicate the observed pattern of oriented ε-Fe_2_O_3_ rods, observed pattern of nonoriented ε-Fe_2_O_3_ rods, and calculated patterns, respectively. (**f**) Magnetization versus external field plot of the ε-Fe_2_O_3_-rod oriented film measured at room temperature along a single in-plane direction.

The electric conductivity of the mesoscopic ε-Fe_2_O_3_ rod was investigated by impedance measurements with a precision impedance analyzer in the frequency range from 40 Hz to 110 MHz using the four-terminal pair method. Figure 3a shows the real (*Z*′) and imaginary (*Z*″) parts of the complex plane impedance. The Cole-Cole circular arc fitting plot, which considers a Gaussian distribution of the conductivity, gives a conductivity value (*σ*) of 9 × 10^−8^ S cm^−1^. Therefore, the mesoscopic ε-Fe_2_O_3_ rod has an insulating character.

The crystal structure of this material is a polar crystal with the *Pna*2_1_ space group. In fact, hybrid functional calculations suggest an electric polarization along the *c*-axis as shown in the charge polarization map of Fig. 3b (see Methods). In this map, the positively and negatively charged areas are attributed to the Fe cations and O anions, respectively.

Additionally, the ferroelectric hysteresis loop was measured in this mesoscopic ε-Fe_2_O_3_ rod (Fig. 3c). The result shows that the mesoscopic ε-Fe_2_O_3_ rod exhibits ferroelectricity, although the remanent electric polarization and the electric coercive field are slightly lower than the reported values for ε-Fe_2_O_3_ nanocrystals deposited on a SrTiO_3_ substrate by pulsed laser deposition^31^. Such a mesoscopic ferrite bar magnet based on a single-magnetic-domain polar-crystal (Fig. 3d) has yet to be reported.

### Thin film based on an oriented ferrite bar magnet

To investigate the magnetic hysteresis loop of the mesoscopic ε-Fe_2_O_3_ rods, we prepared a crystallographically oriented ε-Fe_2_O_3_ film using the following procedure; a dispersion solution containing ε-Fe_2_O_3_ rods, polyurethane, and polyvinylchloride was initially dropped onto a PET film. Then the coated film was dried at room temperature under an external magnetic field of 3 Tesla. The XRD pattern of the obtained film shows one sharp peak of (2 0 0). The orientation degree of the crystallographic *a*-axis in the in-plane direction of the film was evaluated by Rietveld refinement (Fig. 3e). The crystallographic *a*-axis (longitudinal direction) of the ε-Fe_2_O_3_ rod is remarkably well oriented along a single direction in the film plane. The Lotgering factor is 0.96. The magnetic hysteresis measurements using a superconducting quantum interference device (SQUID) show an *H*_c_ value of 25.2 kOe along the *a*-axis at 300 K and a saturation magnetization value of 16.2 emu g^−1^ at 7 T. The *H*_c_ value of 25.2 kOe is the largest among the *H*_c_ values of ε-Fe_2_O_3_ reported to date (Fig. 3f).

### Magnetically controlled non-linear magneto-optical film

**Figure 4:**
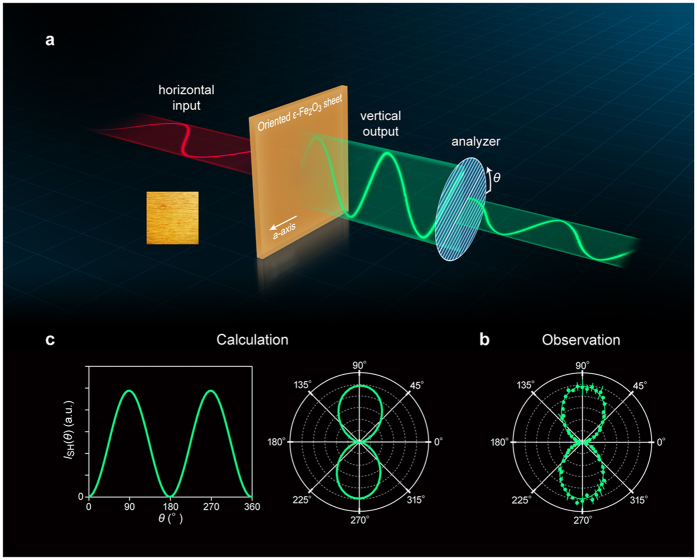
Angular dependence of the non-linear Faraday effect on the oriented ε-Fe_2_O_3_ /resin PET sheet. (**a**) Optical coordinates of the experimental setup of magneto-optical non-linear Faraday effect, where the crystallographic *a*-axis of the ε-Fe_2_O_3_ rod is oriented along the horizontal direction in the sheet. Input light shown in red is horizontally polarized 775 nm fundamental light, and output light shown in green is vertically polarized 388 nm second harmonic light. Left inset shows a photograph of the crystallographically oriented ε-Fe_2_O_3_/resin PET sheet with a size of 1 mm × 1 mm. (**b**) Observed *I*_SH_(*θ*) versus *θ* polar figure. (**c**) Calculated *I*_SH_(*θ*) versus *θ* curve (left) and calculated *I*_SH_(*θ*) versus *θ* polar figure (right) based on the theory of non-linear Faraday effect in *Pna*2_1_ magnetic space group (see Methods).

The correlation between the magnetic polarization along the *a*-axis and the electric polarization along the *c*-axis can induce a non-linear Faraday effect. Therefore, we conducted second-order non-linear optical measurements using a sheet of ε-Fe_2_O_3_ rod/resin on PET. Figure 4a shows the optical coordinates of the measurement system, in which the crystallographic *a*-axis of the ε-Fe_2_O_3_ rod is oriented along the horizontal direction. The sample was irradiated with horizontally polarized incident light (λ = 775 nm femtosecond laser). The rotation angular dependence of the output second harmonic light intensity *I*_SH_(*θ*) in the transmission mode shows that the *I*_SH_(*θ*) value follows sin^2^*θ* curve where it is zero at *θ* = 0°, gradually increases to a maximum at 90°, decreases to zero at 180°, increases again to a maximum at 270°, and finally returns to zero at 360° (Fig. 4b). The clear contrast between light and shade shows a double loop plot in the polar figure; that is, when the ε-Fe_2_O_3_ mesoscopic rod is irradiated with polarized light parallel to the longitudinal direction along the crystallographic *a*-axis, a vertically polarized second harmonic light is emitted (Supplementary Movie 4).

The observed effect can be understood as follows. The second-order non-linear magneto-optical tensor in the *Pna*2_1_ magnetic space group shows that *I*_SH_(*θ*) in the present optical coordinates is expressed as


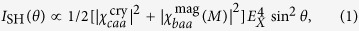


where 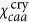 and 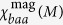 represent the crystallographic and the magnetic terms of the second harmonic susceptibility, respectively. (See Methods for details). The calculated angular dependence agrees well with the observed plots as shown in Fig. 4c.

Tilting the sample sheet by a tilted angle (*ϕ*) of +30° towards the direction of the incident light shifts the maximum and minimum values by +3.3°. On the other hand, a −30° tilt shifts the maximum and minimum values of ISH(*θ*, *ϕ*) by −2.5°. The expected *I*_SH_(*θ*, *ϕ*) and the observed data are shown in Supplementary Information §8 and Supplementary Figures S4 and S5, respectively. If the second harmonic signal only has a crystallographic term, then the absolute value of the ±shift should be the same. Therefore, the difference in the shift angles originates from the magnetic term of 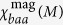, demonstrating that the oriented ε-Fe_2_O_3_ mesoscopic rod/resin on PET is a magnetically controlled non-linear optical rotation sheet.

### Application to an MFM probe on a cantilever

**Figure 5:**
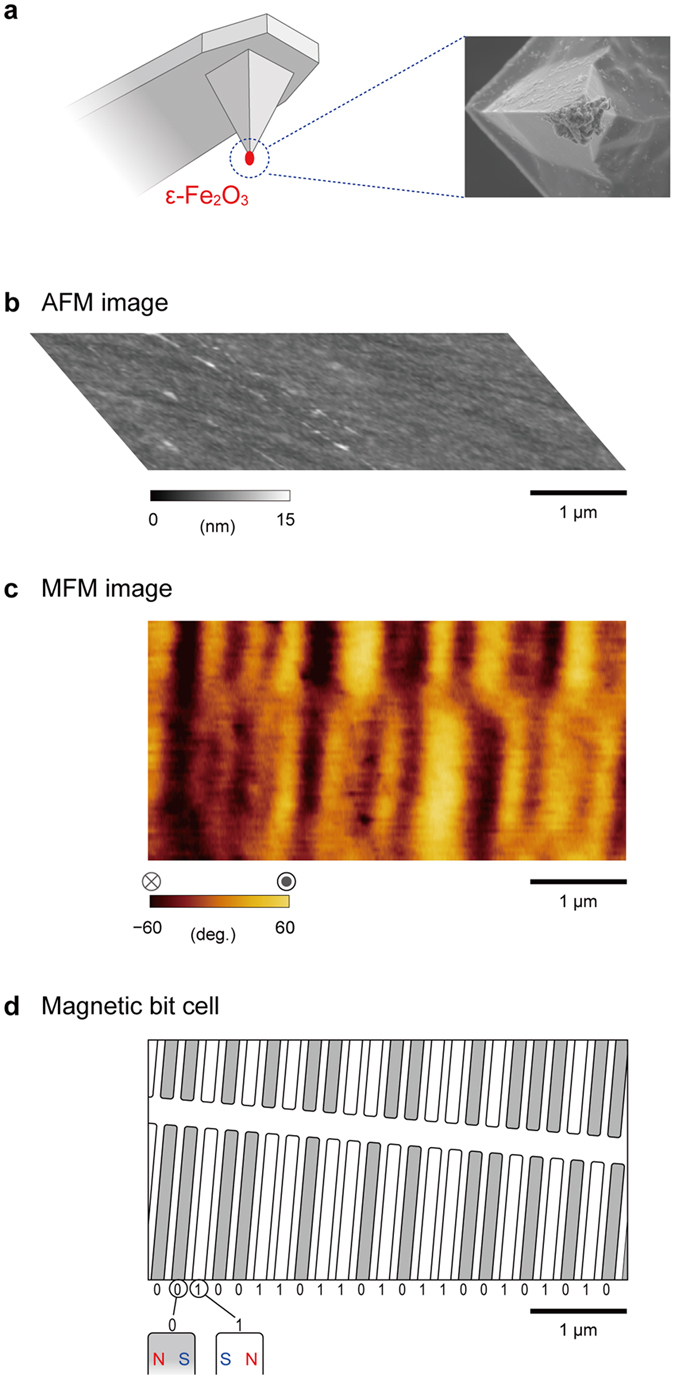
Demonstration of the mesoscopic ε-Fe_2_O_3_ rod as an MFM probe. (**a**) Schematic illustration of the ε-Fe_2_O_3_ rod mounted on a cantilever (left) and SEM image of the cantilever tip (right). (**b**) AFM image of a hard disc medium measured using the ε-Fe_2_O_3_-MFM probe. (**c**) MFM image of a hard disc medium measured using the ε-Fe_2_O_3_-MFM probe. Color scale indicates the vector of the magnetic flux going out of (orange) and into (black) the page, which correpond to the N-pole and S-pole, respectively. (**d**) Schematic image of the magnetic bit pattern for the observed area of the hard disc medium. Gray and white areas denote the magnetic bit cells with the magnetic pole directions as shown in the lower left enlarged illustration, which are expressed as 0 and 1, respectively.

The ε-Fe_2_O_3_ mesoscopic bar magnet has a gigantic *H*_c_ value, indicating that it resists an external magnetic flux. Therefore, it should be useful as an MFM probe^37^. We prepared an MFM probe using an ε-Fe_2_O_3_ rod mounted onto the tip of the non-magnetic cantilever. Figure 5a shows a schematic illustration and SEM image of the ε-Fe_2_O_3_-MFM probe.

Using this ε-Fe_2_O_3_-MFM probe, we measured the magnetic bit cells on a commercially available hard disc, Microdrive of IBM, which is composed of a cobalt magnetic layer. The AFM image of the surface of this hard disc shows that the surface is very flat with a roughness less than 0.4 nm (Fig. 5b). The same area was measured using the ε-Fe_2_O_3_-MFM probe, and we obtained an MFM image with a striped pattern due to magnetic flux leakage from the magnetic bit cells (Fig. 5c). In this MFM image, the black and orange areas indicate the directions of the magnetic flux on the surface of the hard disc medium going out of and into the page, which correspond to the N-pole and S-pole, respectively. The intervals between the stripes vary because the magnetic pole direction of each magnetic bit cell depends on the recorded information. Strong color is observed at the S-S or N-N borders, while the contrast is weak at the N-S or S-N borders. Figure 5d schematically shows the magnetic bit pattern, which is inferred from the observed MFM image. Gray and white areas denote the magnetic bit cells with the magnetic pole directions as shown in the lower left enlarged illustration of Fig. 5d, which are expressed here as 0 and 1, respectively. In the present MFM image, the bit cells are magnetically recorded as the bit pattern of 00100110110101100101010 (Fig. 5d). Hence, the ε-Fe_2_O_3_-MFM probe successfully acquires an MFM image.

The ε-Fe_2_O_3_-MFM probe has the following advantages. (i) The magnetic poles of the ε-Fe_2_O_3_-MFM probe are resistant to a strong magnetic flux from the measured magnetic objects or an applied magnetic field. (ii) Since ε-Fe_2_O_3_ is an insulator, the ε-Fe_2_O_3_-MFM probe does not disorder the magnetization states of the measured magnetic object due to electric current leakage from the cantilever.

## Conclusion

In this paper, we report a mesoscopic single crystal bar magnet composed of ε-Fe_2_O_3_ that withstands strong magnetic fields and electric current flow. Additionally, it does not rust. Typical bar magnets are produced by hot pressing a magnetic powder under an external magnetic field. However, the present type of ferrite bar magnet (i.e., an ideal single crystal ferrite bar magnet with a single magnetic domain structure) has not been previously reported because most ferrite magnets exhibit a weak magnetic anisotropy, preventing a single magnetic domain structure.

Concerning the magnetic anisotropy energy density of ε-Fe_2_O_3_, the value is considered to be 7 × 10^6^ erg cm^−3 ^32, which is much higher than the values for other ferrite magnets. Spectroscopic measurements over a wide frequency range (0.075–750 THz) show that (i) the magneto-optical transition moment of the Faraday effect is along the longitudinal direction of the bar magnet, (ii) the atomic movement of the lowest frequency phonon mode vibrates along the direction of the bar magnet, (iii) bulk magnetization precesses around the longitudinal direction of the bar magnet, and (iv) a non-linear magneto-optical Faraday effect, which depends on the direction of the bar magnet, is observed (Supplementary Movie 5).

We also demonstrate a “magnetization-responsive organic-inorganic composite non-linear optical sheet” as a new application for the ε-Fe_2_O_3_ rod/resin on PET. The mesoscopic ε-Fe_2_O_3_ rod is resistant to the magnetic flux of any magnetic material, and this bar magnet exhibits insulating properties. As an example of an application of ε-Fe_2_O_3_, we demonstrated a cantilever probe for magnetic force microscopy. The “ε-Fe_2_O_3_-MFM probe” has advantages for measuring strong permanent magnets and avoiding turbulence in the image by an electric current due to accidental contact between the probe and the measured magnetic object.

## Methods

### Material

Reverse-micelle solution containing iron(III) nitrate and barium(II) nitrate aqueous solution was mixed with reverse-micelle solution containing ammonium hydroxide. Then tetraethyl orthosilicate was added and stirred to form a silica matrix. The obtained precursor was sintered in air at 980 or 1025 °C. The silica matrix was etched according to the modified technique of our previous method^27^. Details are described in the Supplementary Information.

### Physical measurements

SEM and TEM images were acquired using a JEOL JSM-7001F and JEOL JEM 2000EX, respectively. XRD patterns were measured by a Rigaku Ultima IV and Rigaku SmartLab. AFM and MFM measurements were conducted by a Hitachi High-Tech Science AFM5000II/AFM5300E MFM. For the Faraday effect and Far-IR measurements, a JASCO E-250 magneto-optical meter and a JASCO 6100 spectrometer were used, respectively. The magnetic measurements were carried out using a Quantum Design MPMS SQUID magnetometer. The impedance measurements were conducted with an Agilent 4294A precision impedance analyzer. Ferroelectric property was measured using a TF Analyzer 1000, aixACCT electric polarization hysteresis meter.

### Magneto-optical transition moment calculation

The magneto-optical transition of ε-Fe_2_O_3_ was calculated by Vienna ab initio Simulation Package^38^. The magnetic spins were treated by a spin-polarized model. The Brillouin zone was integrated with 7 × 5 × 5 *k*-mesh and first order Gaussian smearing with a width of 0.1 eV. The magneto-optical transition probabilities of the up-spin and down-spin were obtained from the calculated optical matrix elements, while the sum was calculated using the Gaussian waveform for up-spin → up-spin transitions and down-spin → down-spin transitions.

### Phonon mode calculation

The phonon modes were calculated by the Phonon code^39^ in a Material Design MedeA package. The atomic positions of ε-Fe_2_O_3_ were optimized with an energy cutoff of 400 eV and 3 × 3 × 3 *k*-mesh until satisfying a 10^−5^ eV pm^−1^ force tolerance. The optimized structures were used to calculate the phonon modes of ε-Fe_2_O_3_, which were determined by the direct method implemented in Phonon code with 2-pm displacements using the optimized structures.

### Charge polarization map calculation

The charge polarization map in the unit cell of ε-Fe_2_O_3_ was obtained by screened Coulomb hybrid functional calculation of HSE06^40^. The positive and negative charge distributions were calculated by the difference between the charge distribution of ε-Fe_2_O_3_ and the charges of the Fe and O atoms.

### Magnon (Kittel mode) measurement

The resonance of the mesoscopic ε-Fe_2_O_3_ rod due to Kittel-mode magnon in the millimeter wave region was measured by THz time-domain spectroscopy. A THz pulse wave was generated using a mode-locked Ti: sapphire pulse laser as a source and a dipole type low-temperature-grown GaAs photoconductive antenna as an emitter. The transmitted THz electric field was detected with a bowtie type low-temperature-grown GaAs photoconductive antenna, which was recorded with variable time delays as a temporal waveform in the time domain using a pump-probe system.

### Landau-Lifshitz analysis with interference effect

Kittel-mode magnon spectrum was analysed by considering multiple reflection within the sample. Magnon absorption (*A*) is described by:


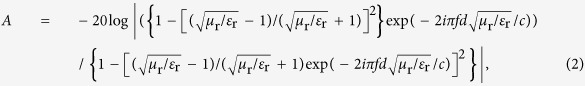


where *d*, *μ*_r_, and *ε*_r_ are thickness, relative magnetic permeability, and relative dielectric constant of the ε-Fe_2_O_3_ pellet sample, respectively, *i* is the imaginary unit, and *c* is the speed of light. *μ*_r_, which is a function of frequency (*f* ), is derived from the Landau-Lifshitz equation^36^,^37^ describing the motion of the magnetization against the intrinsic magnetic anisotropy field of the magnet and the magnetic field of the THz wave;


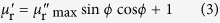



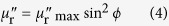



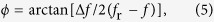


where *f*_r_ is the resonance frequency, 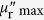 is the maximum value of 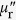, and Δ*f* is the full width at half maximum of 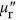 peak.

### Angular dependence of non-linear Faraday effect

Because the sample is magnetized along the magnetic easy axis of the *a*-axis, the magnetic space group of the oriented film of ε-Fe_2_O_3_ is *Pna*2_1_. In this case, the non-zero tensor elements are 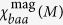, 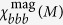, 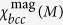, 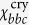, 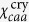, 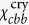, 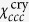, 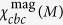, 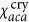, and 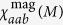, where *a*, *b*, and *c* denote the crystal axes of ε-Fe_2_O_3_. When *θ* is the angle from the horizontal direction, which is also the analyzer angle, the polarization of the output SH light (*P*(*θ*)) is


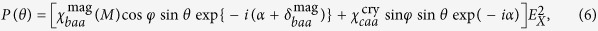


where *X*, *Y*, and *Z* are the coordinates of the measurement system, *φ* is the angle between the *b*-axis (or *c*-axis) of the ε-Fe_2_O_3_ mesoscopic rod and the *Y*-axis (or *Z*-axis) of the optical coordinate, *α* is the phase shift due to light propagation, and 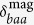 is the phase shift between the magnetic term and the crystallographic term. Thus, *I*_SH_(*θ*) is described as


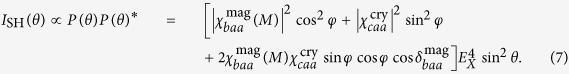


By integrating *I*(*θ*) with respect to *φ* from 0 to 2π, *I*_SH_(*θ*) of the sample with a random orientation in the *bc* plane is expressed as


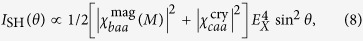


which is proportional to sin^2^*θ*.

## Additional Information

**How to cite this article**: Ohkoshi, S. *et al.* Mesoscopic bar magnet based on ε-Fe_2_O_3_ hard ferrite. *Sci. Rep.* **6**, 27212; doi: 10.1038/srep27212 (2016).

## Supplementary Material

Supplementary Information

Supplementary Movie 1

Supplementary Movie 2

Supplementary Movie 3

Supplementary Movie 4

Supplementary Movie 5
