# Supplementary material for: Mesoscopic bar magnet based on ε-Fe2O3 hard ferrite
Source: Sci Rep. 2016 Jun 7;6:27212. doi: 10.1038/srep27212 (PMC4895237; doi:10.1038/srep27212)
Supplement: Supplementary Information [file srep27212-s1.pdf]

Supplementary Information

Mesoscopic bar magnet based on ε-Fe_2O_3 hard ferrite

Shin-ichi Ohkoshi,[1] Asuka Namai,[1] Takehiro Yamaoka,[2] Marie Yoshikiyo,[1] Kenta Imoto,[1] Tomomichi Nasu,[1] Shizuka Anan,[1] Yoshikazu Umeta,[1] Kosuke Nakagawa,[1] and Hiroko Tokoro[1,3]*

[1] *Department of Chemistry, School of Science, The University of Tokyo, 7-3-1 Hongo, Bunkyo-ku, Tokyo 113-0033, Japan*
[2] *Hitachi High-Tech Science Corporation, Kanagawa Science Park, R&D Business Park Bldg., C-1F, 3-2-1, Sakado, Takatsu-ku, Kawasaki-shi, Kanagawa 213-0012, Japan*
[3] *Division of Materials Science, Faculty of Pure and Applied Sciences, University of Tsukuba, 1-1-1 Tennodai, Tsukuba, Ibaraki 305-8577, Japan*

**§ 1.**	Legends for Supplementary Movies 1–5.		**S2, S3**
**§ 2.**	Detailed synthesis procedure of mesoscopic ε-Fe_2O_3 rods.		**S4**
**§ 3.**	Crystallographic data of the mesoscopic ε-Fe_2O_3 rods.	Table S1	**S5**
**§ 4.**	SEM images of dispersed mesoscopic ε-Fe_2O_3 rods on a carbon plate.	Figure S1	**S6**
**§ 5.**	Dielectric functions of ε-Fe_2O_3 calculated by first-principles calculation.	Figure S2	**S7**
**§ 6.**	Far-IR spectrum and calculated phonon modes spectrum, phonon dispersion, and phonon density of states of ε-Fe_2O_3.	Figure S3	**S8**
**§ 7.**	Frequencies and symmetries of the calculated phonon modes.	Table S2	**S9**
**§ 8.**	Expected second harmonic light intensity with tilted sample.		**S10**
**§ 9.**	Measurement of angular dependent non-linear Faraday effect with tilted sample.	Figures S4,S5	**S11**

§ 1. Legends for Supplementary Movies 1–5.

Supplementary Movie 1:
Faraday spectrum & Magneto-optical transition calculation.
The initial part of this movie shows the observed Faraday spectrum (upper) and the calculated magneto-optical transition probability from the first-principles calculation (lower) of ε-Fe_2O_3. Red sticks, blue sticks, and yellow line indicate the transition probabilities of the up-spin → up-spin transitions, down-spin → down-spin transitions, and their sum, respectively. Next, the unit cell of the ε-Fe_2O_3 crystal structure, which grows into a mesoscopic rod, appears. Then, the electric field (E) of the electromagnetic (EM) wave that is input from the end of the rod parallel to the *a*-axis is converted into rotated elliptical light.

Supplementary Movie 2:
Far-IR spectrum & Phonon mode (lattice vibration) calculation.
The initial part of this movie shows the observed Far-IR spectrum (upper) and the calculated phonon mode spectrum from the first-principles calculation (lower) of ε-Fe_2O_3. Tick marks indicate the calculated frequencies of the phonon modes, and the vibrational symmetry of each mode is shown below the tick marks, e.g., A_1, A_2, B_1, and B_2. Next the atomic movement of the lowest frequency vibrational mode of A_1 symmetry is shown in the ε-Fe_2O_3 unit cell, first in the *bc* plane and then in the *ac* plane. Red and gray balls represent Fe and O atoms, respectively. Finally the vibration mode is shown on the mesoscopic rod.

Supplementary Movie 3:
THz time-domain spectroscopy & Magnon (Kittel mode), Landau-Lifshitz analysis.
The initial part of this movie shows the observed THz absorption spectrum of the mesoscopic ε-Fe_2O_3 rod (upper) with the temporal waveform of the transmitted THz pulse in the inset, which is Fourier transformed to obtain the absorption spectrum. Lower figure is the simulated spectrum obtained from the Landau-Lifshitz analysis. Next, the analyzed magnetic permeability (real (μ') and imaginary (μ'') parts) is shown on the right. The absorption mechanism is explained by the Kittel-mode magnon (natural resonance). The precession of bulk magnetization (magenta arrow) occurs around the magnetic easy-axis by the magnetic field (M) of the EM wave when irradiated in the *a*-axis direction. In the last part of the movie, the phenomenon is shown on the mesoscopic rod.

Supplementary Movie 4:
Angular dependence of non-linear Faraday effect.
This movie shows the angular dependence of the non-linear magneto-optical Faraday effect observed in a sheet of mesoscopic ε-Fe_2O_3 rods. When the sheet is irradiated with horizontally polarized fundamental light (775 nm), a non-linear Faraday effect causes vertically polarized second harmonic light (388 nm). The latter part of the movie shows the analyzer angle (θ) dependence of the output intensity. The lower left shows the theoretical calculation of $I_{SH}(\theta)$ value based on tensor analysis, which is proportional to $\sin^2\theta$. The lower right is the observed data, which agrees well with the calculation.

Supplementary Movie 5:
Spectroscopic studies in a wide frequency range: magneto-optical transition, phonon, magnon, and non-linear Faraday effect.

The initial part of the movie shows the magneto-optical transition of the mesoscopic ε-Fe_2O_3 rod, where electric field (E) of the EM wave that is irradiated from the end of the rod parallel to the *a*-axis is converted into rotated elliptical light. The second part shows the lattice vibration mode of ε-Fe_2O_3, which illustrates the atomic movements of the lowest frequency vibrational mode. The third part shows the Kittel-mode magnon of the mesoscopic ε-Fe_2O_3 rod, where magnetic field (M) of the EM wave induces the precession of bulk magnetization (magenta arrow) around the magnetic easy-axis, which is the crystallographic *a*-axis in the longitudinal direction of the mesoscopic rod. The last part of the movie shows the non-linear magneto-optical Faraday effect, which generates vertically polarized second harmonic light when the ε-Fe_2O_3 rod is irradiated with horizontally polarized incident light.

§ 2. Detailed synthesis procedure of mesoscopic ε-Fe_2O_3 rods.

Mesoscopic ε-Fe_2O_3 rods were synthesized via the following procedure. Two types of reverse-micelle solutions (I and II) were prepared using 38.8 mmol of hexadecyltrimethylammonium bromide, 157 mmol of *n*-butanol, 450 mmol of octane, and 1.3 mol of water. Reverse-micelle I contained 2.9 mmol of iron(III) nitrate and 0.29 or 0.58 mmol of barium(II) nitrate dissolved in the aqueous phase, while reverse-micelle-II contained 133 mmol ammonia dissolved in the aqueous phase. Reverse-micelle I and II were mixed while stirring, and then 31 mmol of tetraethyl orthosilicate was injected. After stirring, the precipitate was collected and washed with chloroform and methanol using a centrifugal precipitator. Then the washed precipitate was calcined at 980 or 1025 ºC for four hours in ambient air. The calcined powder was etched by an aqueous sodium hydroxide solution at 70 ºC, and washed by an aqueous hydrochloric acid solution to remove the silica matrix covering the iron oxide rods.

§ 3. Crystallographic data of the mesoscopic ε-Fe_2O_3 rods.

Table S1. Crystallographic data and atomic positions of the mesoscopic ε-Fe_2O_3 rods obtained by Rietveld refinement of the XRD pattern.

Crystal system	Orthorhombic		
Space group	*Pna*2₁ (No.33)		
a (Å)	5.08923(16)		
b (Å)	8.7858(3)		
c (Å)	9.4766(2)		
V (Å^3)	423.73(2)		
Density (g cm^{-3})	5.01		
Z	8		
R_{wp} (%)	0.85		
S	1.4081		
	x / *a*	*y* / *b*	*z* / *c*
Fe_A	0.315(4)	0.347(3)	0.1561(8)
Fe_B	0.3204(17)	0.0322(8)	0.369(3)
Fe_C	0.3085(16)	0.6606(11)	0.371(2)
Fe_D	0.196(4)	0.845(4)	0.071(2)
O1	0.992(6)	0.003(4)	0.997(5)
O2	0.038(7)	0.337(5)	0
O3	1.018(8)	0.661(6)	0.015(4)
O4	0.166(10)	0.497(4)	0.276(3)
O5	0.166(11)	0.837(6)	0.2720(16)
O6	0.163(13)	0.166(7)	0.246(5)

§ 4. SEM images of dispersed mesoscopic ε-Fe_2O_3 rods on a carbon plate.

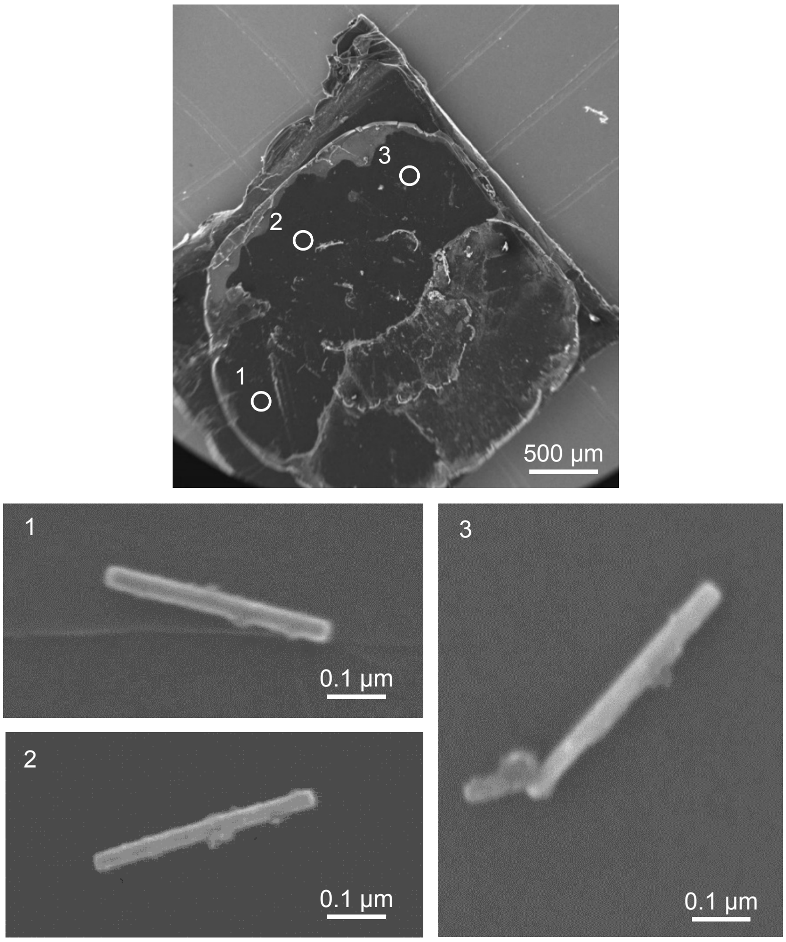

Figure S1. SEM images of mesoscopic ε-Fe_2O_3 rods dispersed on a carbon plate. Areas 1−3, which are marked with open circles (upper), are enlarged in the lower figures.

§ 5. Dielectric functions of ε-Fe_2O_3 calculated by first-principles calculation.

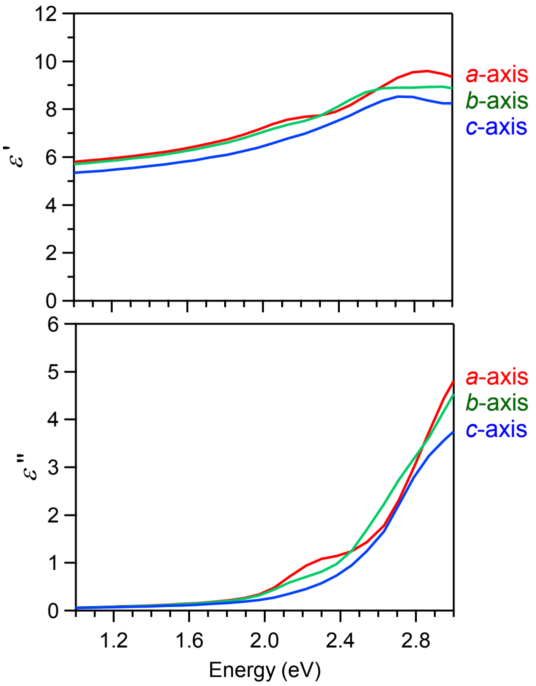

Figure S2. Dielectric functions of ε-Fe_2O_3 calculated by first-principles calculation. Real (upper) and imaginary (lower) parts of the dielectric function (*ε*' and *ε*'') of ε-Fe_2O_3, respectively. Red, green, and blue lines denote the dielectric components for the *a*-, *b*-, and *c*-axes, respectively.

§ 6. Far-IR spectrum and calculated phonon mode spectrum, phonon dispersion, and phonon density of states of ε-Fe_2O_3.

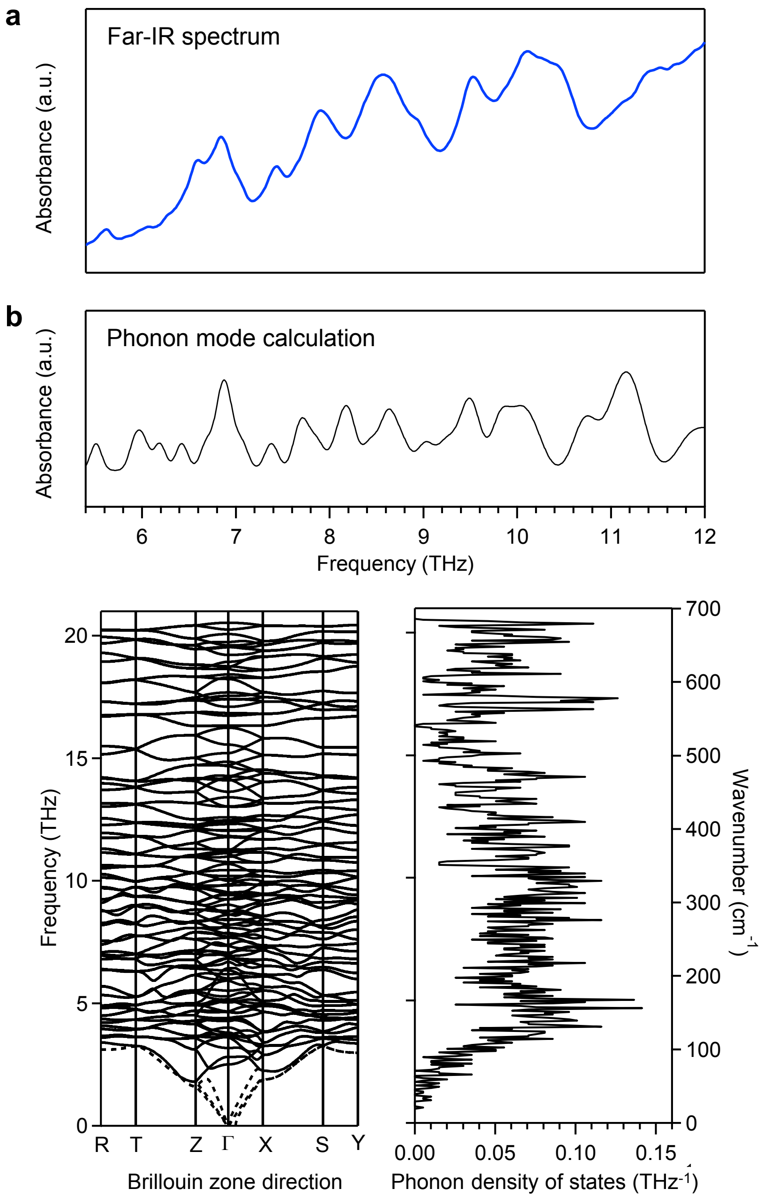

Figure S3. Far-IR spectrum and phonon modes of ε-Fe_2O_3. **a**, Far-IR spectrum of mesoscopic ε-Fe_2O_3 rods and **b**, calculated phonon mode spectrum from the phonon modes calculation of ε-Fe_2O_3. Phonon dispersion (lower left) and phonon density of states (lower right) of ε-Fe_2O_3. Solid and dotted lines of the phonon dispersion indicate optical phonon and acoustic phonon modes, respectively.

§ 7. Frequencies and symmetries of the calculated phonon modes.

Table S2. Optical phonon modes. Energy in frequency and wavenumber, symmetry, and activities of Raman and IR.

No.	THz	cm^{-1}	symmetry	Raman	IR
1	2.51	83.6	A_1	○	○
2	2.82	94.0	A_2	○	—
3	3.18	106.0	B_2	○	○
4	3.61	120.3	A_2	○	—
5	3.62	120.7	B_1	○	○
6	3.62	120.8	A_1	○	○
7	3.73	124.3	B_1	○	○
8	3.80	126.8	A_1	○	○
9	4.01	133.7	A_2	○	—
10	4.27	142.5	B_1	○	○
11	4.34	144.6	B_2	○	○
12	4.40	146.7	A_1	○	○
13	4.65	154.9	B_2	○	○
14	4.65	155.0	A_2	○	—
15	4.67	155.7	A_1	○	○
16	5.04	168.0	A_2	○	—
17	5.10	169.9	B_2	○	○
18	5.14	171.3	A_1	○	○
19	5.16	172.1	B_1	○	○
20	5.28	176.1	A_2	○	—
21	5.51	183.8	B_2	○	○
22	5.92	197.5	B_1	○	○
23	5.96	198.8	A_2	○	—
24	6.02	200.6	A_1	○	○
25	6.19	206.4	B_2	○	○
26	6.34	211.4	A_2	○	—
27	6.42	214.3	B_1	○	○
28	6.69	223.1	B_1	○	○
29	6.83	228.0	A_1	○	○
30	6.84	228.0	B_2	○	○
31	6.91	230.6	B_1	○	○
32	6.92	230.9	A_1	○	○
33	6.99	233.2	A_2	○	—
34	7.06	235.6	B_2	○	○
35	7.35	245.0	A_2	○	—
36	7.38	246.1	B_1	○	○
37	7.67	255.9	B_2	○	○
38	7.73	257.8	B_1	○	○
39	7.85	261.7	A_2	○	—
40	7.87	262.4	A_1	○	○
41	8.09	269.7	B_2	○	○
42	8.20	273.5	B_1	○	○
43	8.20	273.7	B_1	○	○
44	8.40	280.2	A_2	○	—
45	8.43	281.3	A_1	○	○
46	8.61	287.2	B_2	○	○
47	8.63	287.9	B_1	○	○
48	8.75	291.7	A_2	○	—
49	8.77	292.5	B_2	○	○
50	9.02	300.9	A_1	○	○
51	9.24	308.3	B_1	○	○
52	9.25	308.5	A_2	○	—
53	9.42	314.3	B_2	○	○
54	9.47	315.9	A_2	○	—
55	9.52	317.4	A_1	○	○
56	9.53	317.8	A_1	○	○
57	9.81	327.2	B_1	○	○
58	9.85	328.4	B_2	○	○
59	10.00	333.7	B_2	○	○
60	10.08	336.1	A_1	○	○
61	10.20	340.3	B_1	○	○
62	10.44	348.2	A_2	○	—
63	10.69	356.5	A_1	○	○
64	10.77	359.1	B_1	○	○
65	10.98	366.3	B_2	○	○
66	11.12	371.0	B_2	○	○
67	11.13	371.3	A_1	○	○
68	11.20	373.6	A_2	○	—
69	11.24	374.9	A_1	○	○
70	11.29	376.6	B_1	○	○
71	11.78	392.9	A_2	○	—
72	11.83	394.6	B_2	○	○
73	12.04	401.6	B_1	○	○
74	12.07	402.5	A_2	○	—
75	12.31	410.7	B_2	○	○
76	12.43	414.6	A_1	○	○
77	12.44	415.0	B_1	○	○
78	12.47	416.1	A_2	○	—
79	12.60	420.2	A_1	○	○
80	13.02	434.2	B_2	○	○
81	13.03	434.6	A_1	○	○
82	13.41	447.2	A_2	○	—
83	13.63	454.6	A_1	○	○
84	14.10	470.3	B_2	○	○
85	14.15	472.0	A_2	○	—
86	14.21	474.0	B_1	○	○
87	14.28	476.3	B_1	○	○
88	14.69	489.8	A_1	○	○
89	14.71	490.6	B_2	○	○
90	14.86	495.6	A_2	○	—
91	15.21	507.3	B_1	○	○
92	15.56	518.9	A_2	○	—
93	16.25	541.9	B_2	○	○
94	16.33	544.5	B_1	○	○
95	16.34	545.0	A_1	○	○
96	16.74	558.3	B_2	○	○
97	16.85	561.9	A_2	○	—
98	17.10	570.3	A_1	○	○
99	17.41	580.6	A_1	○	○
100	17.42	580.9	A_2	○	—
101	17.55	585.3	B_2	○	○
102	17.69	590.0	B_1	○	○
103	18.23	608.2	A_2	○	—
104	18.30	610.5	B_1	○	○
105	18.42	614.5	B_2	○	○
106	18.64	621.8	A_1	○	○
107	18.72	624.5	B_2	○	○
108	18.76	625.7	A_2	○	—
109	18.94	631.6	B_1	○	○
110	19.22	641.0	A_1	○	○
111	19.53	651.5	B_2	○	○
112	19.55	652.1	A_2	○	—
113	19.61	654.0	B_2	○	○
114	19.75	658.6	B_1	○	○
115	20.07	669.5	A_2	○	—
116	20.26	675.7	B_1	○	○
117	20.53	684.9	A_1	○	○

§ 8. Expected second harmonic light intensity with tilted sample.

We consider the case when the sample sheet is tilted toward the direction of the incident light with a tilted angle of ϕ as shown in the right figure. Second harmonic light intensity is expressed as a function of the rotation angle (θ) and the sample tilted angle (ϕ), $I_{\mathrm{SH}}(\theta, \phi)$, as follows.

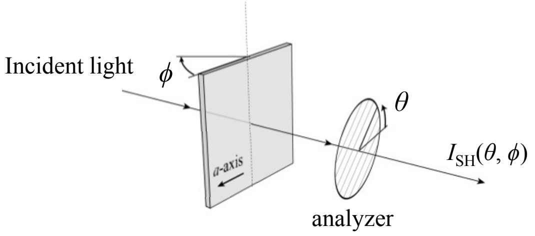

$$I_{\mathrm{SH}}(\theta, \phi) \propto P(\theta, \phi) P(\theta, \phi)^{*}$$

$$= \{ [(4{\chi_{aca}^{\mathrm{cry}}}^2 + 4\chi_{aca}^{\mathrm{cry}}\chi_{caa}^{\mathrm{cry}} + {\chi_{caa}^{\mathrm{cry}}}^2)\sin^2\phi\cos^4\phi + \frac{1}{8}(4{\chi_{bbc}^{\mathrm{cry}}}^2 + 4\chi_{bbc}^{\mathrm{cry}}\chi_{cbb}^{\mathrm{cry}} + {\chi_{cbb}^{\mathrm{cry}}}^2)\sin^6\phi + \frac{5}{8}{\chi_{ccc}^{\mathrm{cry}}}^2\sin^6\phi$$

$$+\frac{1}{2}(4\chi_{aca}^{\mathrm{cry}}\chi_{bbc}^{\mathrm{cry}} + 2\chi_{aca}^{\mathrm{cry}}\chi_{cbb}^{\mathrm{cry}} + 2\chi_{caa}^{\mathrm{cry}}\chi_{bbc}^{\mathrm{cry}} + \chi_{caa}^{\mathrm{cry}}\chi_{cbb}^{\mathrm{cry}})\sin^4\phi\cos^2\phi + \frac{3}{2}(2\chi_{aca}^{\mathrm{cry}}\chi_{ccc}^{\mathrm{cry}} + \chi_{caa}^{\mathrm{cry}}\chi_{ccc}^{\mathrm{cry}})\sin^4\phi\cos^2\phi$$

$$+\frac{1}{4}(2\chi_{bbc}^{\mathrm{cry}}\chi_{ccc}^{\mathrm{cry}} + \chi_{cbb}^{\mathrm{cry}}\chi_{ccc}^{\mathrm{cry}})\sin^4\phi\cos^2\phi + (4{\chi_{aab}^{\mathrm{mag}}}^2 + 4\chi_{aab}^{\mathrm{mag}}\chi_{baa}^{\mathrm{mag}}\cos(\delta_{aab}^{\mathrm{mag}} - \delta_{baa}^{\mathrm{mag}}) + {\chi_{baa}^{\mathrm{mag}}}^2)\sin^2\phi\cos^4\phi$$

$$+\frac{5}{8}{\chi_{bbb}^{\mathrm{mag}}}^2\sin^6\phi + \frac{1}{8}({\chi_{bcc}^{\mathrm{mag}}}^2 + 4\chi_{bcc}^{\mathrm{mag}}\chi_{cbc}^{\mathrm{mag}}\cos(\delta_{bcc}^{\mathrm{mag}} - \delta_{cbc}^{\mathrm{mag}}) + 4{\chi_{cbc}^{\mathrm{mag}}}^2)\sin^6\phi$$

$$+\frac{1}{2}(2\chi_{bcc}^{\mathrm{mag}}\chi_{aab}^{\mathrm{mag}}\cos(\delta_{bcc}^{\mathrm{mag}} - \delta_{aab}^{\mathrm{mag}}) + \chi_{bcc}^{\mathrm{mag}}\chi_{baa}^{\mathrm{mag}}\cos(\delta_{bcc}^{\mathrm{mag}} - \delta_{baa}^{\mathrm{mag}})$$

$$+\frac{3}{2}(2\chi_{aab}^{\mathrm{mag}}\chi_{bbb}^{\mathrm{mag}}\cos(\delta_{aab}^{\mathrm{mag}} - \delta_{bbb}^{\mathrm{mag}}) + \chi_{baa}^{\mathrm{mag}}\chi_{bbb}^{\mathrm{mag}}\cos(\delta_{baa}^{\mathrm{mag}} - \delta_{bbb}^{\mathrm{mag}}))\sin^4\phi\cos^2\phi$$

$$+\frac{1}{4}(\chi_{bbb}^{\mathrm{mag}}\chi_{bcc}^{\mathrm{mag}}\cos(\delta_{bbb}^{\mathrm{mag}} - \delta_{bcc}^{\mathrm{mag}}) + 2\chi_{bbb}^{\mathrm{mag}}\chi_{cbc}^{\mathrm{mag}}\cos(\delta_{bbb}^{\mathrm{mag}} - \delta_{cbc}^{\mathrm{mag}}))\sin^4\phi\cos^2\phi$$

$$+4\chi_{cbc}^{\mathrm{mag}}\chi_{aab}^{\mathrm{mag}}\cos(\delta_{cbc}^{\mathrm{mag}} - \delta_{aab}^{\mathrm{mag}}) + 2\chi_{cbc}^{\mathrm{mag}}\chi_{baa}^{\mathrm{mag}}\cos(\delta_{cbc}^{\mathrm{mag}} - \delta_{baa}^{\mathrm{mag}}))\sin^4\phi\cos^2\phi \quad]\cos^2\theta$$

$$+[{\chi_{caa}^{\mathrm{cry}}}^2\cos^4\phi + \frac{5}{8}{\chi_{cbb}^{\mathrm{cry}}}^2\sin^4\phi + \frac{1}{8}(4{\chi_{bbc}^{\mathrm{cry}}}^2 + 4\chi_{bbc}^{\mathrm{cry}}\chi_{ccc}^{\mathrm{cry}} + {\chi_{ccc}^{\mathrm{cry}}}^2)\sin^4\phi$$

$$+\frac{3}{2}\chi_{caa}^{\mathrm{cry}}\chi_{cbb}^{\mathrm{cry}}\cos^2\phi\sin^2\phi + \frac{1}{4}(\chi_{caa}^{\mathrm{cry}}\chi_{ccc}^{\mathrm{cry}} - 2\chi_{caa}^{\mathrm{cry}}\chi_{bbc}^{\mathrm{cry}})\cos^2\phi\sin^2\phi + \frac{1}{4}(\chi_{cbb}^{\mathrm{cry}}\chi_{ccc}^{\mathrm{cry}} - 2\chi_{cbb}^{\mathrm{cry}}\chi_{bbc}^{\mathrm{cry}})\sin^4\phi$$

$$+{\chi_{baa}^{\mathrm{mag}}}^2\cos^4\phi + \frac{5}{8}{\chi_{bcc}^{\mathrm{mag}}}^2\sin^4\phi + \frac{1}{8}(4{\chi_{cbc}^{\mathrm{mag}}}^2 - 4\chi_{cbc}^{\mathrm{mag}}\chi_{bbb}^{\mathrm{mag}}\cos(\delta_{cbc}^{\mathrm{mag}} - \delta_{bbb}^{\mathrm{mag}}) + {\chi_{bbb}^{\mathrm{mag}}}^2)\sin^4\phi$$

$$+\frac{3}{2}\chi_{baa}^{\mathrm{mag}}\chi_{bcc}^{\mathrm{mag}}\cos(\delta_{baa}^{\mathrm{mag}} - \delta_{bcc}^{\mathrm{mag}})\cos^2\phi\sin^2\phi + \frac{1}{4}(\chi_{baa}^{\mathrm{mag}}\chi_{bbb}^{\mathrm{mag}}\cos(\delta_{baa}^{\mathrm{mag}} - \delta_{bbb}^{\mathrm{mag}}) - 2\chi_{baa}^{\mathrm{mag}}\chi_{cbc}^{\mathrm{mag}}\cos(\delta_{baa}^{\mathrm{mag}} - \delta_{cbc}^{\mathrm{mag}})\cos^2\phi\sin^2\phi$$

$$+\frac{1}{4}(\chi_{bcc}^{\mathrm{mag}}\chi_{bbb}^{\mathrm{mag}}\cos(\delta_{bcc}^{\mathrm{mag}} - \delta_{bbb}^{\mathrm{mag}}) - 2\chi_{bcc}^{\mathrm{mag}}\chi_{cbc}^{\mathrm{mag}}\cos(\delta_{bcc}^{\mathrm{mag}} - \delta_{cbc}^{\mathrm{mag}}))\sin^4\phi \quad]\sin^2\theta$$

$$+[2(2\chi_{aca}^{\mathrm{cry}}\chi_{baa}^{\mathrm{mag}}\cos\delta_{baa}^{\mathrm{mag}} + \chi_{caa}^{\mathrm{cry}}\chi_{baa}^{\mathrm{mag}}\cos\delta_{baa}^{\mathrm{mag}})\sin\phi\cos^4\phi + \frac{3}{2}(2\chi_{aca}^{\mathrm{cry}}\chi_{bcc}^{\mathrm{mag}}\cos\delta_{bcc}^{\mathrm{mag}} + \chi_{caa}^{\mathrm{cry}}\chi_{bcc}^{\mathrm{mag}}\cos\delta_{bcc}^{\mathrm{mag}})\sin^3\phi\cos^2\phi$$

$$+\frac{1}{2}(2\chi_{aca}^{\mathrm{cry}}\chi_{bbb}^{\mathrm{mag}}\cos\delta_{bbb}^{\mathrm{mag}} - 4\chi_{aca}^{\mathrm{cry}}\chi_{cbc}^{\mathrm{mag}}\cos\delta_{cbc}^{\mathrm{mag}} + \chi_{caa}^{\mathrm{cry}}\chi_{bbb}^{\mathrm{mag}}\cos\delta_{bbb}^{\mathrm{mag}} - 2\chi_{caa}^{\mathrm{cry}}\chi_{cbc}^{\mathrm{mag}}\cos\delta_{cbc}^{\mathrm{mag}})\sin^3\phi\cos^2\phi$$

$$-2(2\chi_{aab}^{\mathrm{mag}}\chi_{caa}^{\mathrm{cry}}\cos\delta_{aab}^{\mathrm{mag}} + \chi_{baa}^{\mathrm{mag}}\chi_{caa}^{\mathrm{cry}}\cos\delta_{baa}^{\mathrm{mag}})\sin\phi\cos^4\phi - \frac{3}{2}(2\chi_{aab}^{\mathrm{mag}}\chi_{cbb}^{\mathrm{cry}}\cos\delta_{aab}^{\mathrm{mag}} + \chi_{baa}^{\mathrm{mag}}\chi_{cbb}^{\mathrm{cry}}\cos\delta_{baa}^{\mathrm{mag}})\sin^3\phi\cos^2\phi$$

$$-\frac{1}{2}(2\chi_{aab}^{\mathrm{mag}}\chi_{ccc}^{\mathrm{cry}}\cos\delta_{aab}^{\mathrm{mag}} - 4\chi_{aab}^{\mathrm{mag}}\chi_{bbc}^{\mathrm{cry}}\cos\delta_{aab}^{\mathrm{mag}} + \chi_{baa}^{\mathrm{mag}}\chi_{ccc}^{\mathrm{cry}}\cos\delta_{baa}^{\mathrm{mag}} - 2\chi_{baa}^{\mathrm{mag}}\chi_{bbc}^{\mathrm{cry}}\cos\delta_{baa}^{\mathrm{mag}})\sin^3\phi\cos^2\phi$$

$$-\frac{3}{2}\chi_{bbb}^{\mathrm{mag}}\chi_{caa}^{\mathrm{cry}}\cos\delta_{bbb}^{\mathrm{mag}}\sin^3\phi\cos^2\phi - \frac{5}{4}\chi_{bbb}^{\mathrm{mag}}\chi_{cbb}^{\mathrm{cry}}\cos\delta_{bbb}^{\mathrm{mag}}\sin^5\phi - \frac{1}{4}(\chi_{bbb}^{\mathrm{mag}}\chi_{ccc}^{\mathrm{cry}}\cos\delta_{bbb}^{\mathrm{mag}} - 2\chi_{bbb}^{\mathrm{mag}}\chi_{bbc}^{\mathrm{cry}}\cos\delta_{bbb}^{\mathrm{mag}})\sin^5\phi$$

$$-\frac{1}{2}(\chi_{bcc}^{\mathrm{mag}}\chi_{caa}^{\mathrm{cry}}\cos\delta_{bcc}^{\mathrm{mag}} + 2\chi_{cbc}^{\mathrm{mag}}\chi_{caa}^{\mathrm{cry}}\cos\delta_{bcc}^{\mathrm{mag}})\sin^3\phi\cos^2\phi - \frac{1}{4}(\chi_{bcc}^{\mathrm{mag}}\chi_{cbb}^{\mathrm{cry}}\cos\delta_{bcc}^{\mathrm{mag}} + 2\chi_{cbc}^{\mathrm{mag}}\chi_{cbb}^{\mathrm{cry}}\cos\delta_{cbc}^{\mathrm{mag}})\sin^5\phi$$

$$-\frac{1}{4}(\chi_{bcc}^{\mathrm{mag}}\chi_{ccc}^{\mathrm{cry}}\cos\delta_{bcc}^{\mathrm{mag}} - 2\chi_{bcc}^{\mathrm{mag}}\chi_{bbc}^{\mathrm{cry}}\cos\delta_{bcc}^{\mathrm{mag}} + 2\chi_{cbc}^{\mathrm{mag}}\chi_{ccc}^{\mathrm{cry}}\cos\delta_{cbc}^{\mathrm{mag}} - 4\chi_{cbc}^{\mathrm{mag}}\chi_{bbc}^{\mathrm{cry}}\cos\delta_{cbc}^{\mathrm{mag}})\sin^5\phi$$

$$+\frac{1}{2}(2\chi_{bbc}^{\mathrm{cry}}\chi_{baa}^{\mathrm{mag}}\cos\delta_{baa}^{\mathrm{mag}} + \chi_{cbb}^{\mathrm{cry}}\chi_{baa}^{\mathrm{mag}}\cos\delta_{baa}^{\mathrm{mag}})\sin^3\phi\cos^2\phi + \frac{1}{4}(2\chi_{bbc}^{\mathrm{cry}}\chi_{bcc}^{\mathrm{mag}}\cos\delta_{bcc}^{\mathrm{mag}} + \chi_{cbb}^{\mathrm{cry}}\chi_{bcc}^{\mathrm{mag}}\cos\delta_{bcc}^{\mathrm{mag}})\sin^5\phi$$

$$+\frac{1}{4}(2\chi_{bbc}^{\mathrm{cry}}\chi_{bbb}^{\mathrm{mag}}\cos\delta_{bbb}^{\mathrm{mag}} - 4\chi_{bbc}^{\mathrm{cry}}\chi_{cbc}^{\mathrm{mag}}\cos\delta_{cbc}^{\mathrm{mag}} + \chi_{cbb}^{\mathrm{cry}}\chi_{bbb}^{\mathrm{mag}}\cos\delta_{bbb}^{\mathrm{mag}} - 2\chi_{cbb}^{\mathrm{cry}}\chi_{cbc}^{\mathrm{mag}}\cos\delta_{cbc}^{\mathrm{mag}})\sin^5\phi$$

$$+\frac{3}{2}\chi_{ccc}^{\mathrm{cry}}\chi_{baa}^{\mathrm{mag}}\cos\delta_{baa}^{\mathrm{mag}}\sin^3\phi\cos^2\phi + \frac{5}{4}\chi_{ccc}^{\mathrm{cry}}\chi_{bcc}^{\mathrm{mag}}\cos\delta_{bcc}^{\mathrm{mag}}\sin^5\phi + \frac{1}{4}(\chi_{bbb}^{\mathrm{mag}}\chi_{ccc}^{\mathrm{cry}}\cos\delta_{bbb}^{\mathrm{mag}} - 2\chi_{cbc}^{\mathrm{mag}}\chi_{ccc}^{\mathrm{cry}}\cos\delta_{cbc}^{\mathrm{mag}})\sin^5\phi]\sin\theta\cos\theta \quad \}{E_X}^4$$

§ 9. Measurement of angular dependent non-linear Faraday effect with tilted sample.

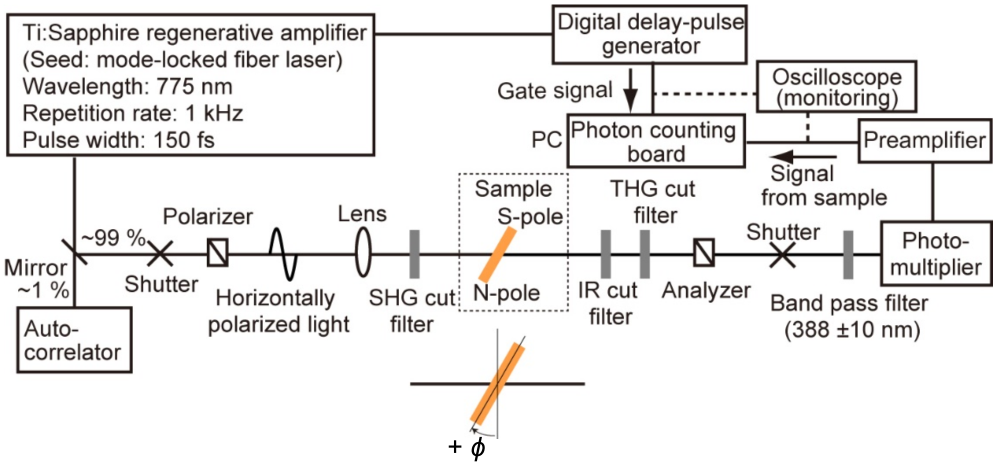

Figure S4. Experimental setup for MSHG measurement. 775-nm light was used as the incident light, and the detection of the second harmonic (SH) light was performed by a photomultiplier tube after passing through color filters according to reference 32. Inset figure shows the top view of the arrangement for the measurements with the tilted sample.

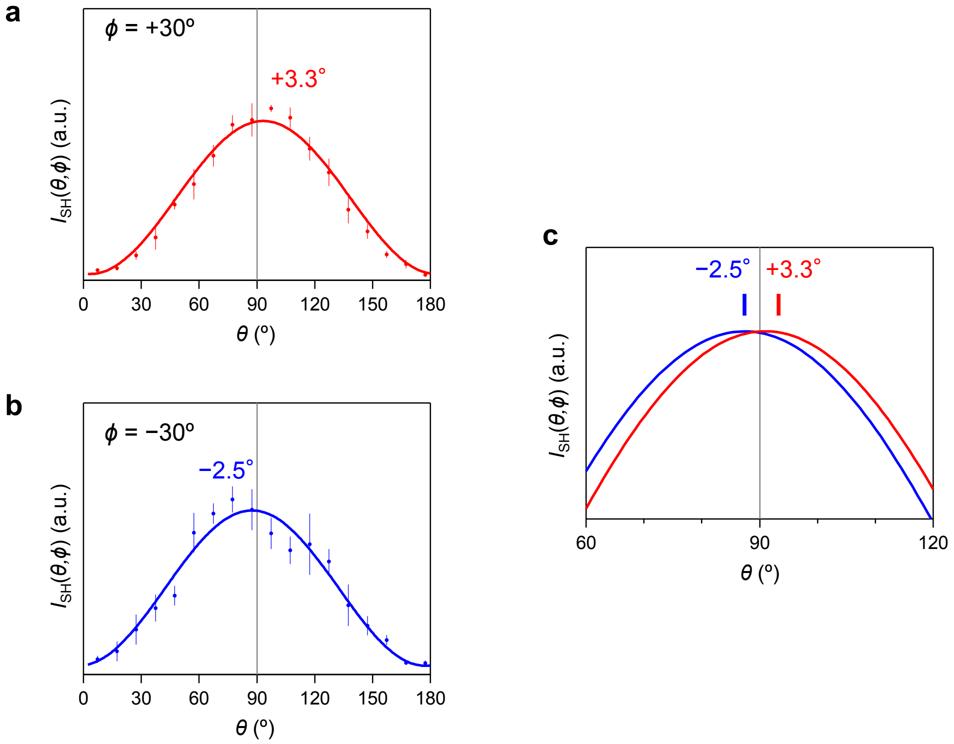

Figure S5. a, $I_{SH}(\theta,\phi)$ versus θ plots for ϕ = +30º. Red dots and red line represent the observed data and the fitted curve, respectively. **b**, $I_{SH}(\theta,\phi)$ versus θ plots for ϕ = −30º. Blue dots and blue line represent the observed data and the fitted curve, respectively. **c**, Fitted $I_{SH}(\theta,\phi)$ versus θ curves for ϕ = +30º (red line) and ϕ = −30º (blue line). The equation of $I_{SH}(\theta,\phi) = a \sin^2\theta + b \sin\theta cos\theta + c \cos^2\theta$ was used, where a, b, and c are the fitting parameters. Gray vertical lines indicates the center positions at θ = 90º, and red and blue tick marks show the maximum positions of the fitted curves for ϕ = +30º and −30º, respectively.
